# Analyzing Metabolic States of Adipogenic and Osteogenic Differentiation in Human Mesenchymal Stem Cells via Genome Scale Metabolic Model Reconstruction

**DOI:** 10.3389/fcell.2021.642681

**Published:** 2021-06-04

**Authors:** Thora Bjorg Sigmarsdottir, Sarah McGarrity, James T. Yurkovich, Óttar Rolfsson, Ólafur Eysteinn Sigurjónsson

**Affiliations:** ^1^School of Science and Engineering, Reykjavík University, Reykjavík, Iceland; ^2^Center for Systems Biology, University of Iceland, Reykjavík, Iceland; ^3^Department of Bioengineering, University of California, San Diego, La Jolla, CA, United States; ^4^The Blood Bank, Landspitali – The National University Hospital of Iceland, Reykjavík, Iceland

**Keywords:** GEM, MSCs, osteogenesis, metabolic reconstruction, adipogenesis, metabolic differences

## Abstract

Since their initial discovery in 1976, mesenchymal stem cells (MSCs) have been gathering interest as a possible tool to further the development and enhancement of various therapeutics within regenerative medicine. However, our current understanding of both metabolic function and existing differences within the varying cell lineages (e.g., cells in either osteogenesis or adipogenesis) is severely lacking making it more difficult to fully realize the therapeutic potential of MSCs. Here, we reconstruct the MSC metabolic network to understand the activity of various metabolic pathways and compare their usage under different conditions and use these models to perform experimental design. We present three new genome-scale metabolic models (GEMs) each representing a different MSC lineage (proliferation, osteogenesis, and adipogenesis) that are biologically feasible and have distinctive cell lineage characteristics that can be used to explore metabolic function and increase our understanding of these phenotypes. We present the most distinctive differences between these lineages when it comes to enriched metabolic subsystems and propose a possible osteogenic enhancer. Taken together, we hope these mechanistic models will aid in the understanding and therapeutic potential of MSCs.

## Introduction

Mesenchymal stem cells (MSCs) originate in the mesenchymal germ layer of the embryo and can be isolated from various adult tissues, including bone marrow, adipose tissue and skeletal tissue to name a few. *In vitro* MSCs are a heterogeneous population of cells that can be expanded and differentiated to chondrocyte, adipocyte and osteocyte lineages (Liu et al., 2006). MSCs have been used as tissue replacements with mixed results but recent studies suggest that their paracrine effect may be of greater clinical importance (Rosenbaum et al., 2008; Duijvestein et al., 2010; Mohyeddin Bonab et al., 2012; Campana et al., 2014; Goldberg et al., 2017; Sigmarsdóttir et al., 2020). The differentiation of MSCs is characterized by chemical and mechanical signals (such as changes to available metabolites, Croitoru-Lamoury et al., 2011; Buravkova et al., 2013) and changing metabolic capabilities (Chen et al., 2008).

This study focuses on MSCs during expansion, early osteogenesis and early adipogenesis because of the potential utility of the cells as immune-modulators (Aggarwal and Pittenger, 2005; Gan et al., 2008; Waterman et al., 2010; Neman et al., 2012; Valencia et al., 2016; Wang et al., 2018; de Castro et al., 2019) and the intricate inverse relationship that seems to exist between adipogenesis and osteogenesis. Osteoporosis is a common and sometimes a severe age related disease condition that can go on undiagnosed until a major fracture occurs. “Malfunctioning” of MSCs in osteoporosis pushes cells toward adipose accretion in the bone marrow at the expense of osteoblast formation – indicating that under this condition MSC behavior is altered and the microenvironment disturbed. A similar shift has been observed to happen under microgravity conditions, as are seen during space flights (Zayzafoon et al., 2004; Pino et al., 2012; Phetfong et al., 2016; Han et al., 2019). Understanding metabolic aspects of this shift may aid in preventing it. Moreover, understanding metabolic differences between MSCs in expansion and in osteogenic differentiation will allow optimization of *in vitro* expansion and initial *in vitro* osteogenic differentiation of MSCs needed for therapeutic techniques to succeed.

Over the past decade as the rate of biological data generation has increased, data analysis and interpretation has become a bottleneck of biological discovery (Yurkovich and Palsson, 2016), necessitating improved modeling and analysis approaches to organize and interpret data (Becker et al., 2007; Thiele and Palsson, 2010; Noronha et al., 2018). By creating a framework to integrate multiple data types (e.g., metabolomics, transcriptomics, proteomics, and genomics), genome scale metabolic models (GEMs) provide a more nuanced understanding of how the cell achieves a given metabolic state (Oberhardt et al., 2009; Thiele and Palsson, 2010; Aurich et al., 2015, 2016; Yurkovich and Palsson, 2016) that can be further tailored to specific physiological conditions using a variety of constraint-based reconstruction and analysis (COBRA) methods (Becker et al., 2007; Orth et al., 2010; Heirendt et al., 2019).

Genome scale metabolic models have been applied to various biological problems including drug resistance and biomarker identification and have been explored using these approaches (Oberhardt et al., 2009; Motamedian et al., 2015; Väremo et al., 2015). Previously, a GEM of MSC metabolism (iMSC1255) was developed (Fouladiha et al., 2015) and used to assess the effects of metabolic environmental changes on chondrogenesis and proliferation (Fouladiha et al., 2018). However, iMSC1255 is limited by only including transcriptomic data from proliferating MSCs and has not considered osteogenesis or adipogenesis explicitly. Furthermore iMSC1255 was based on Recon1 (Duarte et al., 2007), a base human metabolic reconstruction that has been superseded by Recon3 (Brunk et al., 2018). Recon3 includes greater detail of lipid metabolism and glycan metabolism that are known to be key to the processes of differentiation that these models will be used to study. To provide an improved modeling framework to study MSC osteogenic and adipogenic differentiation this paper describes a new set of three models that separately describe the metabolism of expanding MSCs (iMSC_E_1972), osteogenically differentiating MSCs (iMSC_O_1900), and adipogenically differentiating MSCs (iMSC_A_2036). These models provide improved metabolic coverage by using an updated base model from Recon3 (Brunk et al., 2018). The new models were constructed with publicly available lineage specific transcriptomic data, and new metabolomics data produced in house from each lineage. By creating this set of equivalent but separate models, it is possible to create an *in silico* laboratory that helps to design experiments for the cell culture laboratory with a higher probability of success.

Here, we describe three parallel GEMs of MSC metabolism: during expansion, iMSC-E-1972; osteogenic differentiation, iMSC-O-1900; and adipogenic differentiation, iMSC-A-2036. We have benchmarked these models against generic human metabolic functions, the existing model of MSC metabolism, iMSC1255, and known metabolic differences between lineages. We then used these benchmarked models to propose ideas for optimizing osteogenic differentiation of MSCs that will hopefully improve our understanding of the mechanisms underlying these processes.

## Materials and Methods

### Transcriptomic Data Sets

Transcriptomic data were obtained from ArrayExpress (Kolesnikov et al., 2015). Data sets were selected based on their relevance to the experimental conditions described below for collection of uptake and secretion data. The most important considerations having been lineage of differentiation and the length of time since the start of differentiation (7 days). See Table 1 for details of the data sets.

**TABLE 1:** List of data sets used to create the computational models.

*# of set*	iMSC-E-1972	iMSC-O-1900	iMSC-A-2036
1	Samples and Data < E-MEXP-3046 < Browse < ArrayExpress < EMBL-EBI, 2018	Samples and Data < E-MEXP-3046 < Browse < ArrayExpress < EMBL-EBI, 2018^30^	E-MEXP-858
2	E-MEXP-858	E-TABM-318 (“E-TABM-318 < Experiments Matching “‘Mesenchymal Stem Cell’” < ArrayExpress < EMBL-EBI,” n.d.; Ng et al., 2008)	E-TABM-(“E-TABM-318 < Experiments Matching “‘Mesenchymal Stem Cell’” < ArrayExpress < EMBL-EBI,” n.d.; Ng et al., 2008)
3	E-TABM-318	NA	NA

The selected data sets were processed using MATLAB (Mathworks, Natick, Massachusetts, United States). First, the data set IDs were converted to Entrez IDs using either DAVID (Huang et al., 2009; Agarwala et al., 2018) or the chip data from array expression in combination with MATLAB. These numbers were then normalized within each data set and re-scaled so that the magnitude of the range of expression values was like the starting range (all the data sets had maximal expression values of around 1000 to begin with and this range was approximately maintained) allowing the data to be pooled.

### Metabolic Data Collection

Mesenchymal stem cells were obtained from 5 to 6 different donors from LONZA (Basel, Switzerland, donors with National Bioethics committee number VSN19-189). Cells were stored in liquid nitrogen, thawed and seeded in 175cm^2^ flask in basal medium in an incubator at 5% CO2, 37°C and 95% humidity. For experiments cells were seeded in 75cm^2^ flasks at 6000cells/cm^2^. Cells were either grown in basal growth medium or osteogenic differentiation medium. 5000 IU/ml of heparin (LEO Pharma A/Sm Ballerup, Denmark), 1% Penicillin/Streptomycin (Gibco, Grand Island, NY, United States), and 10% Platelet lysate (Platome Reykjavik, Iceland) into DMEM/F12 + Glutamax growth medium (Gibco, Grand Island, NY, United States). This mixture will hereby be referred to as the basal growth-medium. The platelet lysate (PIPL) was centrifuged at 5000rpm/4975g for ten minutes before the supernatant being added to the medium to remove platelet debris and coagulation. The differentiation medium also with the addition of dexamethasone (50 μl, Sigma, Missouri, SL, United States), BMP-2 (50 μl, Peprotech, Rocky Hill, United States), β-glycerophosphate (108 mg, Sigma, Missouri, SL, United States), and ascorbic acid (50 μl, Sigma, Missouri, SL, United States). Cells were used between passage 2 and 6 and care was taken to ensure that at least 5 of the same donors were used for each lineage. Medium was changed on average every 48 h.

Baseline and spent medium samples were assayed for glucose and lactate concentration using an ABL90 blood gas analyzer (Radiometer Medical ApS, Bronshøj, Denmark). In addition, medium samples were analyzed by high pressure liquid chromatography mass spectrometry as described below.

Cell number was determined by trypan blue exclusion and counting in a hemocytometer and an approximate doubling time calculated, this was confirmed with estimates from the literature (Milo et al., 2010). An estimate of dry cell weight was obtained from the literature (Milo et al., 2010) and the changes in metabolite concentration per hour per gram of dry weight were calculated using linear regression in MATLAB. The resulting estimates of uptake and secretion rate of the measured metabolites were applied as constraints to the metabolic model as described below.

### Mass Spectrometry

UPLC-MS Analyses were performed with an UPLC system (UPLC Acquity, Waters, MA, United Kingdom) coupled in line with a quadrupole-time of flight hybrid mass spectrometer (Synapt G2, Waters, MA, United Kingdom) as described in Paglia et al. (2012).

### Model Construction

Model construction was performed using the Constraint-based reconstruction and analysis (COBRA) Toolbox version 3 in MATLAB, 2017b (Mathworks, Natick, MA, United States). The GEM Recon3 (Brunk et al., 2018) was used as a base reconstruction of the global human metabolic network. We further modified the model to more closely represent MSC metabolism (e.g., extracellular bile acid metabolism and drug metabolism) and to include a greater range of glycan metabolism and lipid metabolism (see Supplementary Table 1). The version of Recon3 downloaded from VMH.life (Virtual Metabolic Human n.d.) had already been constrained to be thermodynamically feasible. We used standard quality checks from the COBRA Toolbox (Thiele and Fleming, n.d.) to ensure that the model remained mathematically and biologically feasible as transcriptomic data were integrated.

We constrained the base model using known medium composition, the list of additional metabolites detected by mass spectrometry in basal medium, and data on cell weight and growth using the Metabotools suite within the COBRA Toolbox (Heirendt et al., 2019). This was done separately for proliferating and osteogenically and adipogenically differentiating MSCs. These three media-constrained models were further constrained using the transcriptomic data described above with core reactions those considered important based on the literature (see Supplementary Table 2) increased to maximal expression and the GIMME algorithm implemented in the COBRA Toolbox (Becker and Palsson, 2008). GIMME was selected as it retains the model growth function. This was considered appropriate because during the process of expansion and the initial stages of differentiation modeled here, MSCs are growing. It was ensured that these models remained mathematically and biologically feasible and that core reactions were all included. Those core reactions that were not included were manually added along with reactions to link them into the model. These transcriptomically constrained models then had uptake and secretion constraints added based on mass spectrometry data with minimal relaxation of these added constraints to allow a feasible model (the list of rates can be found in Method labeled Supplementary Data Sheet, see *Rxn_fluxes_for_O_model, Rxn_fluxes_for_E_model*, and *Rxn_fluxes_for_A_model*). Finally, these models were pruned to give fully functional condition specific models. The models were then checked for the inclusion of core reactions and biological feasibility. We validated the three new reconstructions against the original Recon3 model using Memote (Lieven et al., 2020).

The model iMSC1255 was downloaded (Fouladiha et al., 2015) and used for comparison, for some comparisons the constraints on uptake and secretion of metabolites were altered to create three models; expansion, osteogenic differentiation and adipogenic differentiation.

### Model Comparison

Lethal genes and reactions - those essential to produce flux through the biomass reaction - were determined for each model using the relevant functions in the COBRA Toolbox. Random sampling of the solution space for between 50 and 100% of the optimal biomass reaction flux using the “gpSampler” function in the COBRA Toolbox (Schellenberger and Palsson, 2009), in combination with flux variability analysis (FVA) (Gudmundsson and Thiele, 2010) and flux balance analysis (FBA) (Orth et al., 2010) with the biomass function as the objective were used to determine, the range, probability of distribution, and optimal fluxes through all the model reactions in each model. These were all performed in the COBRA Toolbox. To explore possible reactions needed to optimize osteogenesis the relax reactions function was used. Intersect models including the reactions of either osteogenesis and expansion or osteogenesis and adipogenesis were created, the mean random sampled fluxes for the osteogenic model (with zeros for reactions not in that model) were assigned as the target state and the intersect model with expansion or adipogenic bounds was used as the initial state. An alpha 0.99 was used except where this gave only over 100 reactions to relax, in which case 0.75 was used to achieve lists of reactions to relax that were of a length that could be easily explored. Results from these analyses were assessed by subsystem and gene rule using flux enrichment analysis in the COBRA Toolbox in MATLAB.

## Results

Three comparable models of MSC metabolism representing expansion, osteogenesis and adipogenesis were built using publicly available transcriptomic data and parameterized with newly generated metabolomic data. It was shown that these models could recapitulate known metabolic differences among the three cell subtypes and that has allowed the proposal of novel interventions to optimize osteogenesis that will guide future investigations.

### Metabolomics Data Analysis Indicates Functional Difference

In order to parameterize the three models of MSC differentiation extracellular metabolomic data (UPLC-MS) was generated for each cell lineage. The estimates of uptake and secretion rate of the measured metabolites were applied as constraints to the metabolic model, enabling them to be analyzed in the context of previously published data on metabolic network structure as summarized in Recon 3 (add in relevant reference), and MSC transcripts from cells at the relevant stage and lineage of differentiation (add in references for the array express data sets).

A total of 59 unique metabolites were detected across the three cell culture lineages. Of these metabolites, 42 were detected in the expansion samples, 50 were detected in the osteogenic samples, and 44 were detected in the adipogenic samples. Interestingly, some metabolites showed opposite trends in the different cell lineages. For example, asparagine, folate, and fumarate were taken up only in expanding cells. Ascorbate, aspartate (measured only in O), glycerol 2 phosphate, glycl groups, palmitate, histidine, pantothenate, proline and threonine (only measurable in O) were taken up only in osteogenic cells. Oxoproline, acetyl carnitine, adenine, adenosine, biotin, phosphocholine, guanine, phenylalanine, riboflavin, and spermine were taken up only in adipogenic cells (rate lists can be found in Supplementary Material). These data were analyzed by applying them as constraints to the prior knowledge contained in metabolic networks based on Recon 3 and previously published transcriptomics data. This allowed us to leverage prior knowledge to gain greater insights into the likely metabolic changes to MSCs during differentiation than would have been obtained by statistical methods.

### Newly Reconstructed Models Expand Coverage of Metabolic Pathways and Accurately Represent Core Metabolic Fluxes

**FIGURE 1:**
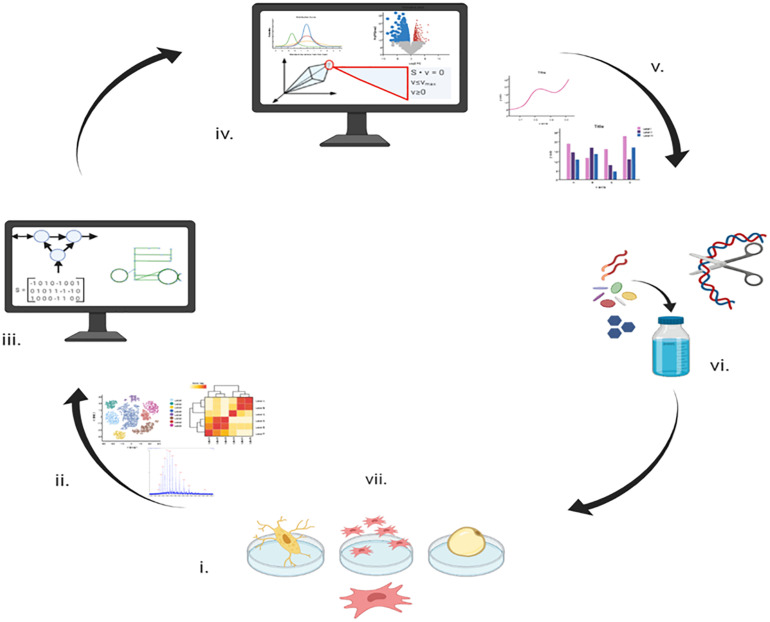
A schematic drawing showing how metabolic modeling can integrate data to contribute to planning future experiments and potential utilization. **Stage (i)** Initial metabolomics and transcriptomics data acquisition from *in vitro* culture. **Stage (ii)** Statistical analysis of metabolomic and transcriptomic data. **Stage (iii)** Reconstruction of the human metabolic network from the literature and previous knowledge. **Stage (iv)** Application of metabolic and transcriptomic data to the metabolic network to produce context specific models. **Stage (v)** Analysis of the differences between the three biological models. **Stage (vi)** Verification of novel findings of the models initially through comparison to the results of published experiments (presented in this manuscript) and then through new experiments. **Stage (vii)** Improved knowledge of the biological system enabling optimized growth and differentiation that may be applied to improve healthcare or industrial outcomes.

Model reconstruction is an iterative process where multiple type of -omic data are combined and results from experiments based on model predictions are used to make future predictions more accurate (Figure 1). As shown in Table 2 the three models were of similar size with a mean of 6517 reactions, 3674 metabolites and 1969 genes, an increase in size of almost 3-fold compared iMSC1255 (2288 reactions, 1850 metabolites, and 1259 genes). The three models had a combined total of 7478 unique reactions, of which 212 reactions were unique to iMSC-E-1972, 151 reactions were unique to iMSC-O-1900, and 457 reactions were unique to iMSC-A-2036. The ability to produce extracellular calcium phosphate—an important function for osteogenic MSCs—was added to the osteogenic model.

**TABLE 2:** Model size at various stages of construction (E) refers to iMSC-E-1972, (O) refers to iMSC-O-1900, and (A) refers to the iMSC-A-2036.

Model	Genes	Reactions	Metabolites
Recon3D	3697	13543	8399
Base Model	2280	9151	4900
GIMME Models *(medium and growth and transcriptomic)*		6693(E) 6061(O) 6933(A)	3792(E) 3472(O) 3884(A)
Corrected Models *(GIMME plus literature)*		6741(E) 6134(O) 7006(A)	3830(E) 3526(O) 3939(A)
Final Models *(Corrected plus metabolomics)*	1972(E) 1900(O) 2036(A)	6600(E) 5975(O) 6876(A)	3740(E) 3428(O) 3855(A)
iMSC1255 model *(used for comparison)*	1259 (E)	2288 (E)	1850 (E)

iMSC-O-1900 contained fewer genes, reactions and metabolites compared to the other two final models, a difference first apparent after implementing the transcriptomic constraint.

The base model, adapted from Recon3D, and the three new iMSC models were assessed using the memote tool (Lieven et al., 2020). The iMSC1255 (Fouladiha et al., 2015) scored 31% overall. This reflected high scores for model consistency but low scores on annotation. iMSC-E-1972 scored 37%, iMSC-O-1900 48% and iMSC-A-2036. Again scores were higher for consistency than annotation, however, annotation was improved. All of the new models include around 2100 stoichiometrically balanced cycles, however, given the size of the models this was unsurprising. The models do not erroneously produce ATP, NAD, or NADP (threshold 1e-10) and a fast leak test from the Cobratoolbox found no leaking metabolites.

None of the newly constructed MSC models showed biologically infeasible behaviors such as creating ATP from water alone or leaking metabolites when uptake was prevented (see Supplementary Table 1). It should be noted that the previously published iMSC1255 generally also fulfilled these criteria but does include some duplicated reactions, possibly due to the treatment of reversible reactions.

The three new MSC models presented here were all capable of producing approximately the correct number of ATP molecules per molecule of both glucose (32 aerobic and 2 anaerobic for all three models) and glutamine (22.5 aerobic and 1 anaerobic all three models) under both aerobic and anaerobic conditions. There were slight differences in theoretical ATP production in the literature and existing models but around 31 from aerobic glucose, 2 from anaerobic glucose, 23 from aerobic glutamine and anaerobic glutamine are common figures obtained in other models including those specifically curated for central energy metabolism (Smith et al., 2017; Mazat and Ransac, 2019). However, the estimates of these values for iMSC1255 were often very different (423 glucose aerobic, 400 glucose anaerobic, 415 glutamine aerobic, and 399 glutamine anaerobic). These large values for ATP production from glucose and glutamine in iMSC1255 were explained by increased activity in the mitochondrial ATPsynthase reaction. The presented new and updated models therefore better captured the energetics of central carbon, fatty acid, and amino acid metabolism.

**FIGURE 2:**
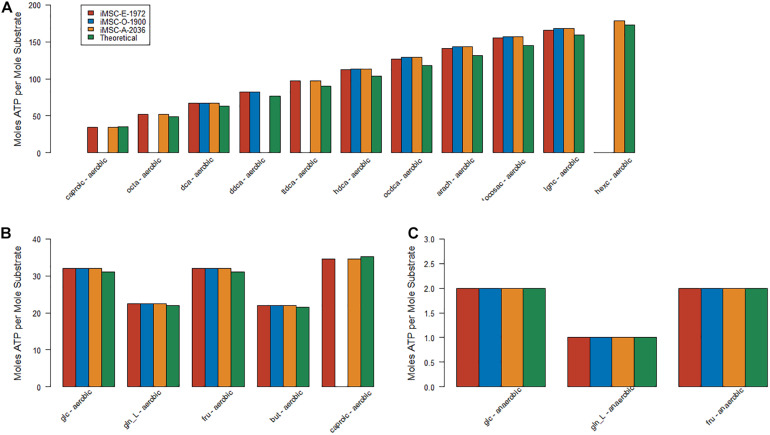
Estimates of optimal fluxes for ATP production from various key carbon sources – **(A)** Aerobic metabolism of the most energy dense substrates **(B)** Aerobic metabolism of the least energy dense substrates **(C)** Anaerobic metabolism.

ATP production from other important carbon sources was also generally close to theoretical values (see Figure 2) in the three new MSC models. The initial values for medium fatty acid composition had a large effect on these values and the differences are likely due to the composition chosen.

The uptake and secretion fluxes of amino acids in the proliferation model and the original iMSC1255 values were compared (see Supplementary Table 2 for details). Nine of the amino acids with uptake/secretion values reported in Fouladiha et al. (2015) were included in the constraints for our model based on our own measurements. These were generally in accordance with the reported constraints. Leucine and lysine fluxes were not based on mass spectrometry measurements, but the range of random sampled fluxes falls at least partially within the same range as the iMSC1255 estimates. Another six amino acids had fluxes in the same predicted direction as iMSC1255 but overestimated the magnitude of uptake or secretion. Two amino acids are predicted as secreted in our random sampling but taken up by iMSC1255.

### Mitochondrial Function Separates Proliferating and Differentiating MSCs and Pentose Phosphate Pathway Flux Differentiates Osteogenesis and Adipogenesis

To determine the quality of the models, model predictions were compared to data about MSC metabolism from the literature. Proliferating, osteogenically and adipogenically differentiating bone marrow derived MSC models were examined and compared to the outputs from the re-constrained versions of iMSC1255 (Fouladiha et al., 2015). The newly produced models matched the data very well for the relative levels of *beta hydroxylase* activity, but iMSC1255 does not include beta hydroxylase reactions. The new models qualitatively predicted differences in flux between proliferating MSCs and one or other of the differentiating lineages but not both in *lactate dehydrogenase, creatine kinase, glyceraldyhyde-3-phosphate dehydrogenase, phosphofructokinase* and *glutathione reductase* with only a slight mis-estimation in the other differentiation lineage. iMSC1255 estimated very similar fluxes across all three models for all of these reactions. *Isocitrate dehydrogenase* appeared to be well estimated in the adipogenic and proliferation models but underestimated in the osteogenic model, iMSC1255 estimated little difference in this reaction.

**FIGURE 3:**
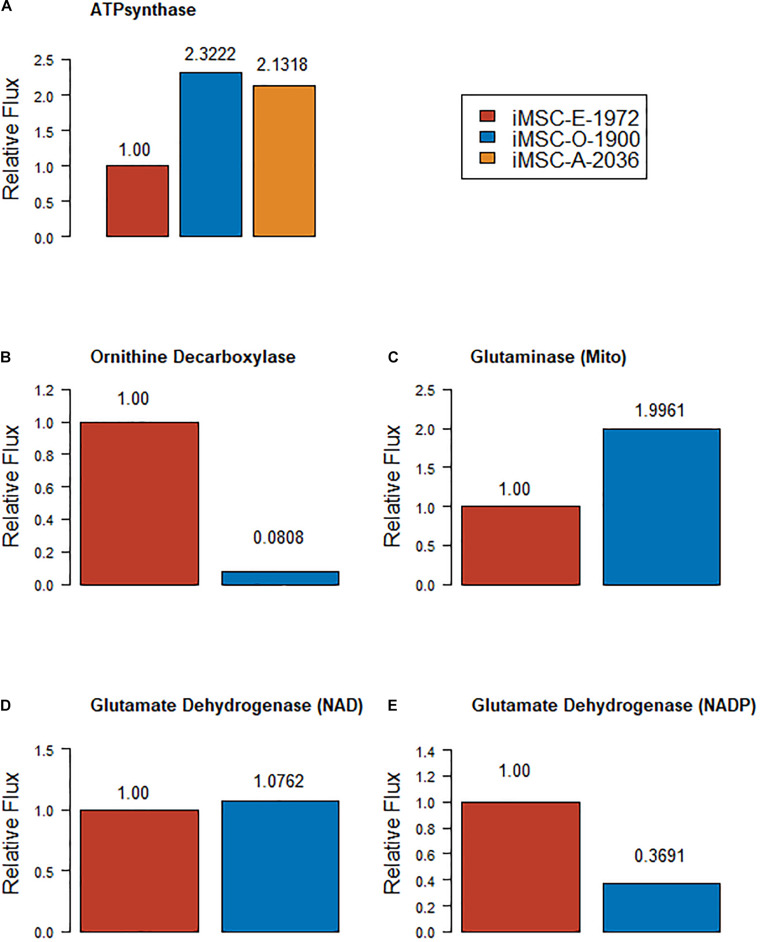
Relative mean fluxes (compared to expansion model) from various reactions – **(A)** ATPsynthase **(B)** Ornithine decarboxylase **(C)** Mitochondrial glutaminase **(D)** NAD dependent glutamate dehydrogenase **(E)** NADP dependent glutamate dehydrogenase.

Various studies have analyzed metabolism in osteogenic and adipogenic differentiation of cells, sometimes in MSCs and sometimes in other cell types (Pattappa et al., 2011; Meleshina et al., 2016; Shum et al., 2016). The new models were compared to several of these studies. Random sampling of the new models with a flux of between 50 and 100% of the biomass function showed that both iMSC-O-1900 and iMSC-A-2036 show increased flux through mitochondrial ATPsynthase, marked as highly significant using the Wilcoxon statistic in MATLAB (ranksum, *p*-Values ∼0 for O vs. E and 5.40 e-81 for E vs. A). Both differentiating models had mean predicted values twice as high as the proliferating model. The minimum values for the iMSC-A and –O were around ten times than in iMSC-E. However, the maximum value in iMSC-E was approximately 10% higher than either differentiating model, indicating that differentiating models showed increased mitochondrial function (Figure 3A). It has also been reported that suppression of ornithine decarboxylase activity increases osteogenesis in human bone marrow MSCs (Tsai et al., 2015). This is supported by the mean flux from random sampling iMSC-E-1972 for ORNDC being greater over 10 times greater than the value of that in iMSC-O-1900 (Figure 3B, *p* ∼ 0 Wilcoxon rank test).

Conversely, it has been shown that suppressing glutaminase activity and the contribution of glutamine to the TCA cycle prevents osteogenesis (Huang et al., 2017; Chen et al., 2019). iMSC-O-1900 showed lower mitochondrial glutaminase (Figure 3C) but higher cytoplasmic glutaminase (not seen in iMSC-E-1972) and overall glutaminase activity more than twice as high as the proliferation model. However, the contribution of glutamine to the TCA cycle via glutamate dehydrogenase (anaplerosis) was slightly higher in the proliferation than the osteogenic model (Figures 3D,E). Other uses of glutamate account for the rest of the osteogenic model’s glutaminase activity. This result indicates that this central area of metabolism may be lacking in sub-cellular location specificity in the models, however, it substantially agrees with the literature on a whole cell basis. Furthermore, it has been shown that the production of kyneurine via indoalamine dioxygenase is increased in osteogenesis compared to proliferation of MSCs (Vidal et al., 2015). A key enzyme in this pathway L-Tryptophan:Oxygen 2, 3-Oxidoreductase (Decyclizing), that produces L formyl-kyneurine is absent from iMSC-E-1972 but present in iMSC-O-1900 (and iMSC-A-2036).

**FIGURE 4:**
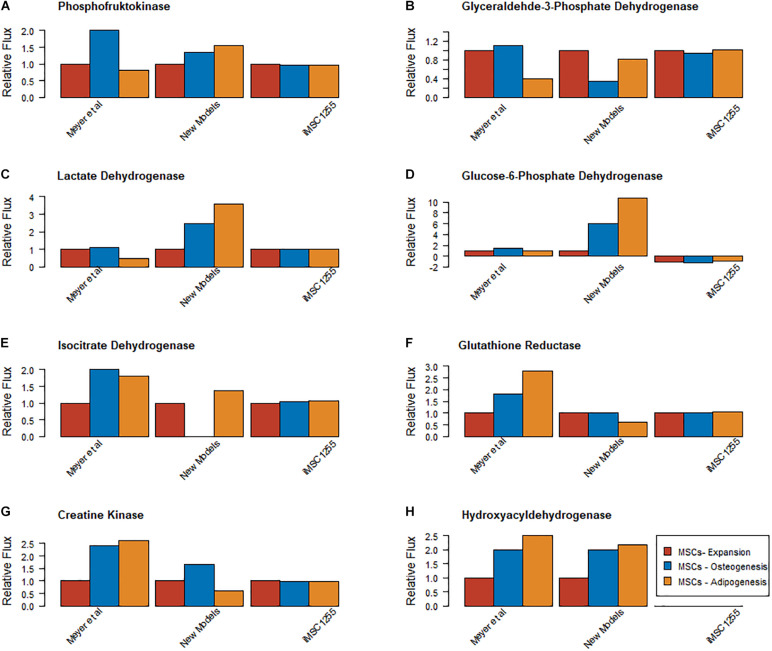
Comparison of relative magnitude of metabolic fluxes found at day 7 of expansion, osteogenic and adipogenic differentiation in Meyer et al. (2018), iMSC1255 er-constrained with MS data collected in Iceland and the new models **(A)** Phosphofructokinase **(B)** Glyceraldyhyde 3 phosphate dehydrogenase **(C)** Lactate dehydrogenase **(D)** Glucose 6 phosphate dehydrogenase **(E)** Isocitrate dehydrogenase **(F)** Glutathione reductase **(G)** Creatine kinase **(H)** Beta hydroxyl acyl dehydrogenase. E, Expansion; O, Osteogenesis; A, Adipogenesis.

A key feature of adipogenesis is the upregulation of glucose 6 phosphate dehydrogenase activity. G6PDH forms 6-Phosphonoglucono-D-lactone (6PGL) and NADPH. 6PGL contributes to the pentose phosphate pathway and therefore the production of nucleotides and reducing potential via NAPDH. NADPH contributes to the synthesis of fatty acids by supplying a reducing agent. This is a key metabolic coupling between glucose and fatty acid utilization and storage (Park et al., 2005). Overall, the formation of NADPH by this reaction was much higher in iMSC-A-2036, around ten times higher than iMSC-E and almost twice that of iMSC-O (Figure 4D). This was partially due to the higher endoplasmic reticulum G6PDH forward activity in iMSC-A-2036 but also due to the presence of some reverse activity being predicted in iMSC-O-1900 and -E-1972.

To assess metabolic differences between MSCs undergoing expansion, osteogenic differentiation, and adipogenic differentiation, Markov chain Monte Carlo (MCMC, Schellenberg et al., 1992) sampling was used to generate a uniform random sampling of the solution space of each of the three the models. A set of predictions for each reaction was obtained and these sets of predictions compared across the three models. Specifically, the fold changes in the means for each reaction were compared, 5-fold change being considered of interest, and a two tailed *t*-test was performed using 1e-12 as a *p*-Value cut-off; see *Metabolic_difference_rxn_pred* in Supplementary Data Sheet for a full list of these reactions.

**FIGURE 5:**
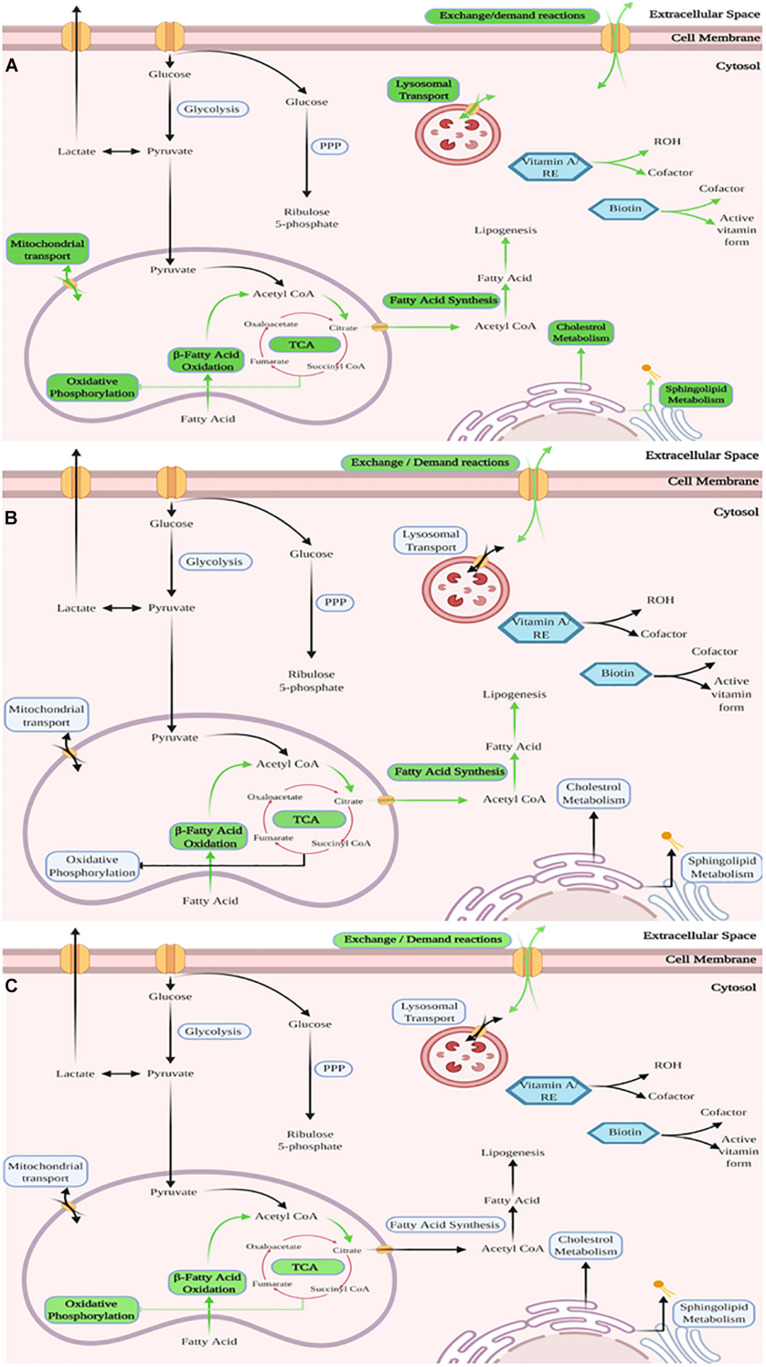
Graphical representation of the enriched subsystems between the cell lineages based on the new reconstructed models – The three figures show in a graphical manner the subsystems (a group of related metabolic reactions representing a specific aspect of metabolism) in each model that were found to contain significantly more changed reactions than other subsystems of the model. Shown is – **(A)** Proliferation of MSCs, with various subsystems identified as enriched. This mirrors expectations as expansion requires energy and synthesis of new material (e.g., cell wall and DNA). **(B)** Adipogenesis of MSCs, with subsystems related to synthesis and breakdown of fatty acids identified as enriched as well as exchange/demand reactions **(C)** Osteogenesis of MSCs, with subsystems related to fatty acid breakdown and TCA-cycle activity identified as enriched as well as exchange/demand reactions. For detailed list with included *p*-Values for each listed subsystem see Table 3. Green arrows and labels indicate enriched pathways.

Reactions in the model were grouped by metabolic subsystem enabling an enrichment analysis to be performed on lists of up regulated reactions (predicted to carry more flux) in each cell type compared to each of the others, shown in Table 3. Enrichment analysis compares which subsytems (areas of metabolism) show more reactions that are up regulated compared to the expected distribution of changes if all changes occurred by chance across the whole of metabolism. This demonstrates those areas of metabolism that are more important to one lineage than another. This enrichment analysis showed that the areas of metabolism that are significantly more active in proliferating cells than in either of the differentiating cell types include biotin metabolism, vitamin A metabolism, sphingolipid metabolism, fatty acid oxidation and fatty acid synthesis as well as mitochondrial transport and exchange/demand reactions. This upregulation showed higher activity in areas of metabolism related to fatty acid signaling (vitamin A and sphingolipids). Sphingolipid metabolism may also indicate the need for new cell membranes. In adipogenic differentiation, fatty acid oxidation and as would be expected fatty acid synthesis were over represented in increased activity along with exchange/demand reactions. Fatty acid oxidation and exchange demand reactions were over represented in increased activity reactions in osteogenesis compared to the other pathways studied, see Figure 5.

**TABLE 3:** Showing subsystems that have a significantly overrepresented (adjusted *p*-value < 0.05) number of more active reactions in the relevant model compared to the other differentiation lineage in the case of osteogenesis/adipogenesis or compared to the two differentiation models in the case of expansion.

**Expansion**			
*Adjusted p-Value*	*Enriched set size*	*Total set size*	*Groups Increased*
0.000254	98	1368	Exchange/demand reaction
0.001831	65	961	Fatty acid oxidation
0.003348	9	242	Cholesterol metabolism
0.003348	9	240	Fatty acid synthesis
0.003348	1	105	Transport, lysosomal
0.003348	25	453	Transport, mitochondrial
0.006098	3	133	Sphingolipid, metabolism
0.011893	7	185	Bile acid synthesis
0.017589	11	47	Vitamin A metabolism
0.019096	5	12	Biotin metabolism
**Osteogenesis**			
*Adjusted p-Value*	*Enriched set size*	*Total set size*	*Groups Increased*
7.09E-07	12	961	Fatty acid oxidation
8.6E-06	28	1368	Exchange/demand reaction
**Adipogenesis**			
*Adjusted p-Value*	*Enriched set size*	*Total set size*	*Groups Increased*
1.64E-05	26	1368	Exchange/demand reaction
0.000253	16	961	Fatty acid oxidation
0.012651	1	240	Fatty acid synthesis

### Model-Driven Experimental Design for Engineering Osteogenesis

Metabolic modeling allows the generation of hypotheses about means of optimizing one cell lineage over another. To propose means of improving osteogenesis desirable for regenerative medicine, combined models of either expansion-and-osteogenesis or adipogenesis-and-osteogenesis were created. Two of each of these models were produced, one representing the constraints on reactions from each lineage with other reactions blocked. These models were then subjected to relaxation analyses.

The results of relaxing the model to find a solution intermediate between either the expansion model or the adipogenic model and the osteogenic model produced lists of key reactions to modify to move towards osteogenesis (see *Relaxed_rxn_lists*, Supplementary Data Sheet, for the more extensive lists required for full transition). The transport reactions to relax to move from expansion to osteogenesis were transport from extracellular to cytosol of serotonin, urea and 13-Docosenoic Acid, in all case the changes moved these metabolites from being taken up to being either not active or in the case of 13-Docosenoic Acid to being secreted. To transform from adipogenic to osteogenic by modifying transport reactions Bilirubin Beta-Diglucuronide uptake should be prevented along with the blocking of leukotriene C4 reduced glutathione transport, thromboxane B2 transport, leukotriene E4 transport and Mono (Glucosyluronic Acid) Bilirubin transport while inward transport of citrate and ribosomal transport of bilirubin should be encouraged. The non-transport reactions (including demand and exchange reactions) to alter to encourage osteogenesis from expansion are: ‘EX_cspg_e[e],’ ‘KAS8,’ ‘r0797,’ ‘RE0344C,’ ‘RE0577C,’ ‘RE0578C,’ ‘RE1845C,’ ‘EX_aicar[e],’ ‘EX_gudac[e],’ ‘EX_stcrn[e],’ ‘EX_Lhcystin[e],’ ‘EX_mal_L[e],’ ‘MYELIN_HSSYN,’ and ‘DM_myelin_hs[c].’ These reactions include amino acid uptake and formation of lipid and glyco lipids. The equivalent reactions for encouraging osteogenesis from adipogenesis are: ‘EX_retfa[e],’ ‘EX_mal_L[e],’ ‘EX_taur[e],’ and ‘DM_na1[r].’ Again, this includes alterations to the necessary amino acids as well as fatty acid retinol involved in developmental signaling. The relaxation required of truly non-transport reactions to bring the expansion model close to the osteogenic model are ‘KAS8,’ ‘r0797,’ ‘RE0344C,’ ‘RE0577C,’ ‘RE0578C,’ and ‘RE1845C.’ These reactions are all involved in fatty acid and CoA metabolism and are generally moving from being active (sometimes in the reverse direction) to being less active or inactive. The equivalent relaxation (with an alpha value of 0.75) produced no necessary changes from adipogensis to osteogenesis however with and alpha of 0.9 ‘3SALACBOXL,’ ‘CBPPer,’ ‘GGNG,’ ‘GLBRAN,’ ‘GLGNS1,’ ‘KAS8,’ ‘LCYSTCBOXL,’ ‘r0060,’ ‘IDL_HSSYN,’ ‘IDL_HSDEG,’ ‘HMR_3422,’ and ‘HMR_6647.’ These reactions become more active or move from a negative to a positive direction while ‘LPS3e,’ ‘NDP7er,’ ‘PCHOL2LINL_HSPLA2,’ and ‘PE203_HSPLA2’ more from the reverse direction to inactivity. Many of these reactions are glycosylating enzymes likely accounting for the alterations in the cells’ outer glycan layer between these two states.

## Discussion

The driving factor behind this study was to improve understanding of the metabolic underpinnings of MSC differentiation with hopes that that will enable further application of them in regenerative medicine. To achieve that three metabolic networks were reconstructed from varied -omics and experimental data and compared. The models achieved to recapitulate key metabolic phenotypic features characteristic for each cell state, to showcase the major metabolic differences and to propose a possible way to improve osteogenesis.

### Measurements of Metabolism

The metabolomic data used in the reconstruction of the presented models was specifically generated and analyzed in order to parametrize the models in accordance to very specific experimental conditions known to the authors to influence the cells in a specific desired manner. The mass spectrometry data shows differences in metabolism between the 3 lineages of cells. These changes in the extracellular milieu support different metabolism during cell growth and differentiation of MSCs. In order to better understand how these extracellular changes reflected intracellular metabolic changes during MSC expansion and differentiation, these data were analyzed in the context of changes in gene transcription within their respective metabolic models.

### Reconstruction of Metabolic Models for Three States of MSCs

Three constraint-based models of MSC metabolism (iMSC-E-1972, iMSC-O-1900, and iMSC-A-2036 describing expansion, osteogenesis, and adipogenesis, respectively) were reconstructed, from the global human metabolic network (Recon3), publicly available transcriptomic data and metabolomic data generated here. These models largely share reactions (2.0 - 6.1% of total reaction number are unique to a model), an expected result considering the transcriptomic data seem to have had the strongest effect on inclusion/exclusion of reactions as demonstrated by showing the biggest change in the number of reactions included in each model (Table 2). These transcriptomics data, and the metabolomics data, represent MSCs at day 7 after first exposure to differentiation medium. Day 7 is still considered as an early stage of the two differentiation processes that take between 21 and 28 days, respectively, and increased lineage differences are expected to be seen at later time points, however, there was insufficient data available for those later time points in a suitable format at the time that this project began. The publicly available transcriptomic data used to generated these models had lower coverage of metabolic genes for the osteogenic state, resulting in a smaller number of reactions included in this model (iMSC-O-1900) when compared to the other two (iMSC-E-1972 and iMSC-A-2036). The osteogenic transcript was missing any call, present or absent for 5.19% of base model genes whilst proliferation and adipogenesis were missing information for only 2.35 and 2.38%, respectively. These models can be continually upgraded and expanded using specifically generated RNAseq data from cells collected at later stages of osteogenesis and adipogenesis in order to further improve the predictive capabilities of the models. The use of RNAseq data in the place of microarray-based data is also likely to improve the coverage of enzyme expression data.

#### Verification of Metabolic Function in Comparison to iMSC1255

This comparison is in some ways limited to broad comments as the enzyme activity assays in the paper presented by Fouladiha et al. (2015) were performed to determine maximal activity in cell extracts, not fluxes in living cell situations as simulated by the models.

The new MSC models all pass key metabolic sanity checks and manage an almost 3-fold increase in metabolic coverage compared to iMSC1255, roughly a proportional increase to that between the base model and Recon1. This similarity suggests retention of similar level of cell specificity compared to iMSC1255 despite the great increase in metabolic coverage. However, the models reported here are able to more correctly capture the production of ATP from various substances and are additionally able to perform more metabolic functions compared to the previously existing MSC model, iMSC1255 (originally reconstructed for cells exclusively in the state of proliferation). One of the areas of metabolic function that the models presented in this paper are more adept at in comparison to iMSC1255 is fatty acid metabolism. This is illustrated by the example of iMSC1255 lacking beta hydroxylase activity (Figure 4), but the activity of beta hydroxylase has been shown to be present in MSCs, as well as its much smaller number of human functions assessed by the COBRA Toolbox (Supplementary Table 1).

As fatty acid metabolism is key to adipogenesis, the additional detail of beta hydroxylase inclusion in the new presented models will likely prove important to the utility of their predictions. The increased coverage of fatty acid and lipid metabolism that is seen in them is in large part due to the updated base reconstruction used for the model’s reconstruction (i.e., using Recon 3 instead of Recon 1). Additionally, when the iMSC1255 model was constrained using the same metabolomic data sets as were used to reconstruct the presented three new MSC models, the new models (iMSC-E-1972, iMSC-O-1900 and iMSC-A-1972) were better able to match the results of Meyer et al. (2018) compared to iMSC1255 (Figure 4). This indicates that by specifically constraining the models with transcriptomics data from various differentiation lineages the models created are better able to show the differences in metabolism between the different lineages.

### Verification of Lineage Specific Metabolic Functions

A good qualitative fit between known metabolic differences between the three lineages studied and the relationships between the predicted fluxes in the three new models was observed. Key metabolic features, such as the production of ATP changing in osteogenesis, are reflected in the differences between the models, indicative of a realistic reconstruction reflecting expected biological behavior. Differences in amino acid metabolism, ornithine and to a lesser extent glutamine and tryptophan, are shown between osteogenesis and expansion in ways that reflect known differences in metabolism during osteogenic differentiation.

G6PDH expression is known to be key to producing NADPH for fatty acid synthesis and overall activity of this enzyme is greater in the adipogenic model than the other two cell lineage models, an observation suggesting that these models reflect this aspect of metabolism well. However, comments on the activity of G6PDH in relation to this paper are complicated by the reversibility of this reaction and the presence in the models of multiple forms of this enzyme. In iMSC1255, the flux is only predicted to be in the reverse direction, creating NADP not NADPH. This does not agree with the previous consensus, our models suggest a mix, specifically our adipogenic model favors NADPH production in accordance with the literature (Park et al., 2005; Melis et al., 2013; Lee et al., 2017; Meyer et al., 2018). These contradictions might suggest that this may be either premature or reflective of the limitations of measuring activity in an isolated enzyme.

Overall, these results demonstrate that this key area of metabolism is well reflected in the models which gives a good ground for building the iMSC-O-1900 model into the later and potentially more metabolically diverse later stages of osteogenesis.

### Observation of Main Metabolic Differences Between the Models

The groups of metabolic reactions that are upregulated in each of the lineages reflect the changes in function of cells during expansion and differentiation. The metabolic subsystems upregulated in expansion are varied, reflecting the necessity of the cells for varied reaction activity to be able to produce cell biomass, membranes, and nucleic acids in order to grow. On the other hand, the changes in adipogenesis are more concentrated around fatty acids and fatty acid synthesis. This is to be expected, especially the increased activity of fatty acid synthesis, which is a key feature of adipocytes. The areas of metabolism with the most concentrated changes in osteogenesis is fatty acid oxidation, which reflects increased oxidative metabolism in the differentiating cells – a phenomenon previously reported by multiple studies (Chen et al., 2008; Pattappa et al., 2011; Buravkova et al., 2013; Shum et al., 2016). All of the cell lines show changes to the exchange reaction activity, which may partially be due to exchange reaction alterations that happen during the model reconstruction process but since many of these observed differences reflect actual measured differences this is unlikely to be problematic.

### Proposed Method to Increase Osteogenesis

The results of the relaxation of reactions between adipogensis or expansion and osteogenesis suggest that alterations in the need for malate are changed, with either reduced uptake or a switch to secretion being observed in both cases. Fatty acid and lipids, particularly those involved in signaling (i.e., retinoic acids and fatty acids) and those involved in cell membranes and glycosylation (i.e., myelin_HS) are altered as well. These are key metabolites for the rearrangement of cell membranes and trans-membrane glycoproteins as well as cell signaling and therefore seem plausible as metabolic markers of differentiation. One of the reactions highlighted is the need to increase transport of citrate from the extracellular space to the cytosol. It has been shown that growing cells on citrate rich materials encourages osteogenesis (Wang et al., 2013; Irizarry et al., 2017), this match with the literature is encouraging. This discovery regarding citrate in combination with the enrichment of the exchange/demand reactions subsystem also suggests that other transporters may be interesting targets for future investigation when it comes to searching for new ways to increase osteogenesis of MSCs.

## Conclusion

We present three new genome-scale metabolic models of MSC metabolism, representing expansion, osteogenesis and adipogenesis differentiation. These newly reconstructed models are increased in scope compared to previous models of this cell type both in terms of the coverage of multiple lineages in models produced specifically for two new lineages and with the models due to usage of a new and much improved base human metabolic reconstruction (Recon3). We computed a variety of metabolic phenotypes, demonstrating that the models presented here accurately represent qualitative and quantitative cellular characteristics and important differences between the cell types. Having validated these models, we used them for model-driven experimental design with the goal to optimize *in vitro* osteogenesis. One of predicted solutions, the citrate transporter, concurs with a previously identified target which encourages further possibilities of similar predictions. Through the use of mechanistic models such as are presented here, we provide a blueprint for the application and engineering of regenerative medicine therapies where promising therapeutics like MSCs can be made more efficient and attainable.

## Data Availability Statement

The data presented in the study are deposited in the MetaboLights repository (https://www.ebi.ac.uk/metabolights/MTBLS2844), accession number MTBLS2844.

## Ethics Statement

The studies involving human participants were reviewed and approved by the National Bioethics committee number VSN19-189. Written informed consent for participation was not required for this study in accordance with the national legislation and the institutional requirements.

## Author Contributions

TS and SMcG designed the study, analyzed the data, and drafted the manuscript. JY, ÓR, and ÓS designed the study and reviewed the manuscript. All authors gave their final approval of the submitted version.

## Conflict of Interest

The authors declare that the research was conducted in the absence of any commercial or financial relationships that could be construed as a potential conflict of interest.
