# Supplementary material for: Analyzing Metabolic States of Adipogenic and Osteogenic Differentiation in Human Mesenchymal Stem Cells via Genome Scale Metabolic Model Reconstruction
Source: Front Cell Dev Biol. 2021 Jun 4;9:642681. doi: 10.3389/fcell.2021.642681 (PMC8212021; doi:10.3389/fcell.2021.642681)
Supplement: Supplementary file 1 [file Data_Sheet_1.docx]

**Supplementary Material**

**Tables**

**Table S1**. Summary of biological feasibility testing

| Test | Base Model | iMSC-E-1972 | iMSC-O-1900 | iMSC-A-2036 | iMSC 1255 |
| --- | --- | --- | --- | --- | --- |
| Metabolite leaks? | 0 | 0 | 0 | 0 | 0 |
| Water -> ATP? | NO | NO | NO | NO | NO |
| Water + H2O -> ATP? | NO | NO | NO | NO | NO |
| ATP -> BIOMASS? | NO | NO | NO | NO | NO |
| Duplicated reactions present? | 0 | 0 | 0 | 0 | 6(0.26%) |
| Reversed demand reactions? | 0 | 0 | 0 | 0 | 0 |
| Key human functions (460)? | 419 | 359 | 334 | 381 | 134 |

**Table S2. Comparison of amino acid uptake and secretion with Fouhilda et al 2015.** Flux values for amino acid uptake/secretion from the expansion model and the values reported for iMSC1255 in the 2015 paper by Fouladiha et al

| Amino Acid Name | Min Theo | Min Exp | Mean Exp | Max Exp | Max Theo |
| --- | --- | --- | --- | --- | --- |
| EX_ala_L[e] | -0,4017 | 355,218 | 958,287 | 997,797 | 0,0969 |
| EX_arg_L[e] | -0,5731 | 0,21537 | 0,26561 | 0,2664 | 0,0458 |
| EX_asn_L[e] | -0,1723 | -22,2531 | -20,314 | -5,5697 | 0,017 |
| EX_asp_L[e] | -0,0291 | 59,7183 | 435,36 | 850,196 | -0,0179 |
| EX_cys_L[e] | -0,0174 | 0,15634 | 132,634 | 664,603 | 0,0907 |
| EX_glu_L[e] | -0,0032 | 0,00724 | 0,00732 | 0,01747 | 0,0962 |
| EX_gln_L[e] | -0,588 | 0,54767 | 0,63738 | 0,6405 | -0,1295 |
| EX_his_L[e] | -0,0283 | -0,0576 | 0,35283 | 0,599 | -0,0038 |
| EX_ile_L[e] | -0,0462 | -0,00795 | -0,00794 | -0,00508 | 0,0005 |
| EX_leu_L[e] | -0,0451 | -0,00852 | -0,00848 | -0,00505 | -0,0089 |
| EX_lys_L[e] | -0,0201 | -0,00435 | -0,00005 | -0,000000024 | -0,004 |
| EX_met_L[e] | -0,007 | 0,01966 | 0,04192 | 0,05249 | -0,001 |
| EX_orn[e] | -0,0163 | -643,36 | 86,1324 | 644,061 | 0,7115 |
| EX_phe_L[e] | -0,0215 | 0,01037 | 0,01403 | 0,01408 | -0,007 |
| EX_pro_L[e] | -0,0143 | -0,33356 | -0,21809 | 1,6289 | 0,0975 |
| EX_ser_L[e] | -0,045 | 123,015 | 495,134 | 813,817 | -0,01 |
| EX_thr_L[e] | -0,0153 | -0,01711 | -0,01651 | 0,01894 | -0,0053 |
| EX_tyr_L[e] | -0,0254 | 150,284 | 541,721 | 857,894 | -0,0076 |
| EX_val_L[e] | -0,0472 | -0,00272 | -0,00271 | 0,00049 | -0,0211 |
| EX_gly[e] | -0,0109 | 215,349 | 622,261 | 931,578 | 0,168 |
